# Properties Evaluation of the Welded Joints Made by Disk Laser

**DOI:** 10.3390/ma14082002

**Published:** 2021-04-16

**Authors:** Ján Viňáš, Janette Brezinová, Henrich Sailer, Jakub Brezina, Miroslav Sahul, Pavlo Maruschak, Olegas Prentkovskis

**Affiliations:** 1 Department of Technology, Materials and Computer Aided Production, Faculty of Mechanical Engineering, Technical University of Košice, 040 01 Košice, Slovakia; jan.vinas@tuke.sk (J.V.); henrich.sailer@tuke.sk (H.S.); jakub.brezina@tuke.sk (J.B.); 2 Department of Welding and Joining of Materials, Faculty of Materials Science and Technology in Trnava, Slovak University of Technology in Bratislava, 917 24 Trnava, Slovakia; miroslav.sahul@stuba.sk; 3 Department of Industrial Automation, Ternopil Ivan Puluj National Technical University, 46001 Ternopil, Ukraine; maruschak.tu.edu@gmail.com; 4 Department of Mobile Machinery and Railway Transport, Vilnius Gediminas Technical University, Plytinės g. 27, LT-10105 Vilnius, Lithuania; olegas.prentkovskis@vilniustech.lt

**Keywords:** laser, welding, dual phase steel, heat treatment, transformation-inducted plasticity steel

## Abstract

The process of laser welding of sheets of HSLA (high-strength low-alloy steel), DP600 (dual-phase steel) and TRIP steels was investigated. A weld was successfully made in a double-sided hot-dip galvanized sheet with a thickness of 0.78–0.81 mm using a laser power of 2 kW per pass without any pretreatment of the weld zone. Microstructure studies revealed the presence of martensitic and ferritic phases in the weld zone, which could be associated with a high rate of its cooling. This made it possible to obtain good strength of the weld, while maintaining sufficient ductility. A relationship between the microstructural features and mechanical properties of welds made in the investigated steels has been established. The highest hardness was found in the alloying region of steels due to the formation of martensite. The hardness test results showed a very narrow soft zone in the heat affected zone (HAZ) adjacent to the weld interface, which does not affect the tensile strength of the weld. The ultimate tensile strength of welds for HSLA steel was 340–450 MPa, for DP600 steel: 580–670 MPa, for TRIP steel: ~700 MPa, respectively, exceeding the strength of base steels.

## 1. Introduction

Laser welding technology is used in various sectors. The laser welding thermal cycle is usually faster than conventional arc technologies, leading to smaller weld widths and heat affected zones. The use of laser technology started after many studies were conducted, however, their first results were not satisfactory. First CO_2_ laser generation could only be used for base materials heating as their melting their performance was not sufficient. In 1971, for the first time the “keyhole” (KD) effect was achieved. Nowadays, laser technologies are widely used in combination with modern computer technology mainly in the areas like mechanical engineering, energy, electrical engineering, medicine, and the army. In mechanical engineering, laser technologies in conjunction with robotization enabling 5-dimensional movement to make the greatest development mainly in the automotive industry where they are most often used for engraving, heat treatment of materials (hardening), cutting and especially welding. Nowadays, car manufacturers such as Audi, Mercedes, BMW, Volvo, Volkswagen, and Škoda use laser technology to increase productivity, product quality and competitiveness [1]. One of the most effective measures to reduce fuel consumption and minimize air pollution is decreasing the weight of vehicles, while maintaining the high level of strength, reliability, and safety. It is known that decreasing the car weight by 10%, as a rule, reduces gas consumption by about 5% [2]. The results of such studies have caused an increased interest in the use of light metal alloys of aluminum and magnesium in the automotive industry. At the same time, non-ferrous metals used in the manufacture of car body parts increase the cost of the car. This necessitates the search for other technical solutions and materials. One of the alternatives is using ultra-high-strength steels (UHSS), primarily, transformation-induced plasticity (TRIP) steels, and dual-phase (DP) steels. These steels have a relatively low cost, high strength, and good shape transformation qualities. Excellent mechanical properties of TRIP steel mainly contribute to the ferrite matrix for ductility, bainite for strength, and retained austenite for uniform elongation produced by martensite transformation from austenite when subjected to external tensile stress. This combination of strength and ductility allows thinner sheets of TRIP steel to be used in the manufacture of vehicles. As a result, the structural strength of vehicles is maintained and sometimes even enhanced. In addition to reducing the weight of the car, its technological properties are improved [3,4,5,6]. This paper summarizes the research findings on three grades of steel currently used in the manufacture of body parts for middle class passenger cars.

### 1.1. Weldability of Progressive Steel Sheets for the Automotive Industry

Today, a significant number of car manufacturers use modern high-strength steels. In the event of a car accident or impact loading, they get a significant part of energy to be used for plastic reshaping of the structure. These steels include, first of all, HSLA (high-strength low-alloy), DP (dual-phase) and TRIP (transformation-induced plasticity) steels [7,8,9]. Of these, the most promising are dual-phase (DP) steels used in the manufacture of such elements as pillars and roof arches that ensure passenger safety during a vehicle rollover. Dual-phase steels consist of two phases (martensite and ferrite), which provide for a tensile strength of about 1 GPa in the presence of optimized morphological ratios and fine grain sizes. At the same time, while this steel meets most of the technological criteria for materials used in the manufacture of vehicle safety elements, its welding is problematic. Any welding is known to change a balanced distribution of phases in the fusing and heat-affected zones, causing the occurrence of micro- and macro-stress concentrators. In particular, it is shown in [10] that laser beam welding (LBW) and electrical resistance welding (ERW) of steel DP980 cause a significant amount of martensite to be formed in the fusing zone (FZ) due to its rapid cooling. In addition, a significant decrease in ductility is observed in welded joints in the presence of a martensitic structure. This reduces the technological possibilities of further reshaping of such sheets, including stamping [11]. The authors of [11] also draw attention to the soft zone within the structure of the heat affected zone (HAZ), where necking occurs long before the material attains boundary deformation. A soft zone is located far from the fusion line (FL) in HAZ as a result of the martensite base material (BM) temper. One of the methods for solving this problem is induction heating at LBW [12]. This makes it possible to overcome the problems associated with the significant structural non-uniformity of welds in modern high-strength steels. Induction heating allows reducing the cooling rate of FZ and HAZ, and provides for the formation of less brittle phases; in particular, bainite. The same method can be used to reduce the amount of martensite in welded joints of steel DP980 [13]. DP 980 is currently a dual phase steel with increased strength applied in the production of car body components.

DP steels have a number of physical and mechanical properties, which make them suitable for the manufacture of car bodies, but, as in the previous case, a number of problems associated with the process of their welding remain unresolved. The presence of highly alloyed additives and metastable phases in the microstructure, as well as a zinc coating on the surface of automobile steel sheets [14], complicates the technology of their welding. The gradient of steel properties in the heat affected zone (HAZ) is also a serious problem for welded structures made of DP steels, which requires additional study [15,16]. Softening in the heat-affected zone of DP steels may lead to a local decrease in hardness, in some cases, to values below the hardness of the base metal (BM). This, in turn, reduces the load-bearing capacity, primarily, the ultimate strength and fatigue life of welded structures made of DP steels [17,18,19,20,21,22]. An increase in the welding speed reduces the intensity of softening, which makes it possible to create welded joints with a tensile strength close to that of BM [23]. One of such approaches is fiber laser welding (FLW) with a very-high speed of welding, which makes it indispensable for LWB [24,25,26,27]. Laser welded structures with joints have a durability comparable to that of BM [28]. At the same time, our recent research has shown that the fatigue life of DP steel welded with fiber laser can be reduced by about 30% compared to BM. In addition, stress concentration in any pore or concavity formed in the weld may cause the initiation of a crack and its further propagation to softer areas [16]. Concavity and softening in the HAZ reduces the fatigue life of welded joints in DP steels [16,17,18,19,20,29]. As a rule, concavity is formed due to the ejection or displacement of metal from the weld pool during welding [29] and depends on its parameters [30,31]. However, metal ejection from the weld pool may also occur during welding when the liquid phase is unstable. This is especially true for the vapor/liquid interface perpendicular to the laser beam at high welding speeds. Reducing the strength and optimizing the welding speed can be one of the options for improving the weld quality [32,33]. However, as emphasized above, an excessive reduction of speed causes an increase in the softening degree of the HAZ, which, in turn, leads to a decrease in the fatigue life and deformation properties of welds. Laser welding of sheets is performed mainly in the form of butt joints. They are considered one of the most difficult joints in laser welding, since the narrow beam and high speed do not allow the beam to leave the joint [33,34], which creates additional difficulties in optimizing welding technology. Low speed causes overheating of welded materials, intensive evaporation of molten metal from the steam-gas channel. The width of the weld metal as well as the width of the HAZ increase. In the wide HAZ range, the grain size increases, which reduces the advantage of laser welding [35]. The coarse-grained structure causes a reduction in the strength properties of welded joints. Increasing the velocity without changing the welding power reduces the isotherm, reduces the volume of molten weld metal in the weld channel, reduces the efficiency of the shielding gas atmosphere, reduces the weld width, and also narrows the HAZ. Excessive increase in welding speed can lead to non-welds. Lack of time for the escape of vapors from the steam-gas channel can cause a significant porosity of the weld metal, which is reflected in a decrease in the strength properties of welded joints. Increasing the welding speed can cause a reduction in the thickness of the weld [36,37].

TRIP (transformation-induced plasticity) steels are believed to be in high demand in the automotive industry. They have an excellent combination of strength and ductility. According to Cui et al. [38], passive safety of car bodies is currently provided, as a rule, by elements made of TRIP steel. According to Khedkar et al. [39], TRIP steels are actively used in the manufacture of vehicle structures that provide safety, such as crossbars, longitudinal beams, reinforcements, sills and bumper reinforcements. According to Baluch et al. [40], new grades of TRIP steel significantly surpass other structural materials in their strength and technological characteristics and will be used for a long time in the designs of both existing and future cars. According to Bhattacharya [41], TRIP steels are, first of all, modern advanced high-strength steels (AHSS), which make it possible to realize significant uniform elongation due to the occurrence of phase transformations caused by plastic deformation. TRIP steels contain many micro components, such as ferrite, bainite, martensite and retained austenite. These micro-structural components help reduce the rebound effect during the stamping of automotive parts and induce martensite upon plastic deformation. Dan et al. [42] argue that the effect of induced plasticity results from the martensitic transformation of metastable austenite, which is realized during strain hardening. It prevents localization of strain by increasing the uniform elongation of the steel [43].

Thus, laser beam welding offers a unique combination of high speed of joining sheet materials and provides for the accuracy and low deformation in the area of the welded joint compared to traditional resistance spot welding [44,45,46]. This is especially true for AHSS (advanced high-strength steels). However, due to an increased carbon concentration (from 0.1% to 0.3%) and the presence of a number of alloying elements (Mn, Si, Al, Cr and Mo), the welding of these steels, in comparison with low-carbon high-strength low-alloy steels (HSLA), requires the welding technology currently in use to be modified. The main technological problem in laser welding of such steels is the tendency of joints to form cold cracks due to high cooling rates and, as a consequence, high hardness, and low ductility of joints. Another common problem is cracking in the joint plane. This, as a rule, is associated with an increased content of carbon and alloying elements, which account for the tendency of AHSS to form martensitic microstructures [47,48,49,50] of high hardness.

High-strength steels are prone to solidification cracks in the weld metal. Solidification cracks initiate in the mushy zone as a result of thermal stresses/strains that are generated due to solidification shrinkage and thermal contraction. The stresses/strain are transmitted when there is an appreciable degree of coherency in the mushy zone. The liquid phase is still present in the form of films separating the grain boundaries [51,52]. At this stage, the solid fraction is typically greater than 0.94 [53]; any opening at the grain boundary is difficult to fill with the remaining liquid due to low permeability. Usually, the condition is further aggravated by the presence of undercooled liquid (extended solidification temperature range) due to the solute enrichment in the liquid, ahead of the solid-liquid interface [54]. If sufficient thermal strains are present, cracking occurs at the grain boundaries where liquid films act as a brittle phase. In this respect, hot tearing refers to the tearing up of the liquid film(s). The reasons for the formation of solidification cracks in welds of high-strength steel types are different from the cracks arising in laser welds in Al alloys used in automotive production, which is documented in works [55,56,57]. Where their origin is influenced by several factors.

The hardness of DP steel (in HAZ) is usually below 400 HV [58], while in TRIP steels, it ranges from 400 to 550 HV, depending on the carbon content [47,49,59]. The greatest hardness is typical for TRIP steels with the addition of Si [60]. The hardenability and hardness of steels can decrease after replacing silicon with aluminum. A significant number of non-metallic inclusions in the melting zone is also a difficult problem. There is a risk of obtaining a soft zone near the melting line as a result of the excessive stabilization of ferrite [61,62]. This problem is partially solved by using TRIP steels, in which silicon is partially replaced by aluminum. The total concentration of these elements should be not less than 1.5 PBW for the retained austenite to stabilize [60,63].

The elongation of TRIP steel is expected to be related to the preserved stability of austenite, which is determined by the carbon content and the morphology and grain size of austenite and the morphologies of other microstructural components [64]. The most important factor is the austenitic carbon content [65]. According to research, the volume fraction of austenite retained among these parent metals is higher in Al-alloy steel. This steel contains less carbon than Si-alloy steel. By mass balance, the carbon content of the Al-alloyed retained austenitic steel is lower, meaning that it reduces the stability of austenite during deformation (i.e., leads to almost complete transformation to martensite at low levels of deformation) and also reduces uniform elongation [66,67]. In the case of Al-alloy steel, the melting zone shows small changes in elongation. Despite the presence of retained austenite. According to previous research [68,69], austenite retained in the form of a film between bainitic ferrite subunits and as one piece can contribute to the TRIP effect by stress transformation. A very important parameter of the transformation effect on ductility is the stability of austenite. This is determined mainly by the size and composition of austenite particles, especially the carbon content [64,69,70]. As already explained in connection with the testing of base metals, it has been suggested that high strain rate testing with strain rate range 10^−1^ to 10^2^ s^−1^ may limit the gradual transformation of austenite retention to martensite, and as a result, the contribution of strain-induced transformation to uniform elongation or complete elongation may be less than in quasi-static testing with strain rate range 10^−5^ to 10^−1^ s^−1^ [71,72,73]. It can be said that there should be elongation effects on the strain rate for steels with a significant TRIP effect, which are found in these base metals. An important reason for the relative lack of effect of strain rate on ductility in this Al-alloyed weld metal is likely to be associated with the formation of low carbon retained austenite and the resulting very-low-stress stability. Thus, the expected martensitic transformation occurs too early in quasi-static testing. As a result, it contributes little to the overall ductility at low or high strain rates. Therefore, the efficiency of the TRIP phenomenon in this Al alloy steel weld metal is too low to be significant.

### 1.2. The Aim of the Current Investigation

The aim of the current research is microstructural characterization of laser welds and destructive tests to verify welded joints quality. To analyze in detail the microstructure in different zones (fusion zone—FZ, heat-affected zone—HAZ, transitional zone—TZ, and base metal—BM) and, especially, to identify retained austenite, it was necessary to use combined light microscopy methods (LM) and scanning electron microscopy (SEM). The metallographic sample preparation started with sectioning cold-mounted samples in an epoxy resin by using an abrasive cutter. Grinding was performed using 320 grit and 600 grit SiC papers. Further grinding on finer abrasive papers (1000 grit, or 1200 grit) was found to be unnecessary. The most important step in the sample preparation was the coarse polishing step, which was preferably performed on a napless cotton cloth with a 6-micron diamond paste. Polished samples, etching in 3% nital (solution HNO_3_) were used to identify the microstructure. Metallographic observations at a magnification of 50× and 500× were carried out with a Keyence VHX 5000 light microscope. Tesla BS SEM using back-scattered electrons (BSE) revealed morphological details of microstructural constituents. Observations were performed on nital-etched samples at an accelerating voltage of 20 kV. Chemical composition of test materials was verified by Belec Compact Port spectrum chemical analyzer.

Microhardness measurements were performed across different areas by Shimadzu HMV-2 microhardness tester, by means of the Vickers method according to EN ISO 6507-1 and applying a load of 980.7 mN (HV0.1). The indentations were positioned at regular intervals of 150 μm. The hardness distribution in heat-affect zone (HAZ), fusion zone and base materials are observed. To obtain the average microhardness value 5 measurements were made at each point. However, since the study was phenomenological in nature, a deeper statistical analysis was not carried out. Static tensile test was performed on ZWICK Z 400 (ZwickRoell GmbH and Co. KG, Ulm, Deutschland), according to EN ISO 6892-1.

## 2. Materials and Methods

This paper presents the results of experimental investigations into the three types of steels most commonly used in the automotive industry, in particular, the production of car bodies and their components. In order to unify the description of the experiments, specimens made from double-sided galvanized sheet HSLA steel with a thickness of 0.81 mm were designated with the letter A. Specimens made of 0.8 mm thick double-sided hot-dip galvanized sheet of DP 600 steel were designated with the letter B, and specimens cut out of galvanized sheet of high-strength TRIP steel with a thickness of 0.78 mm were designated with the letter C. The mechanical properties and chemical composition of the steels are given in Table 1 and Table 2, respectively.

### Disk Laser Welding

**Figure 1:**
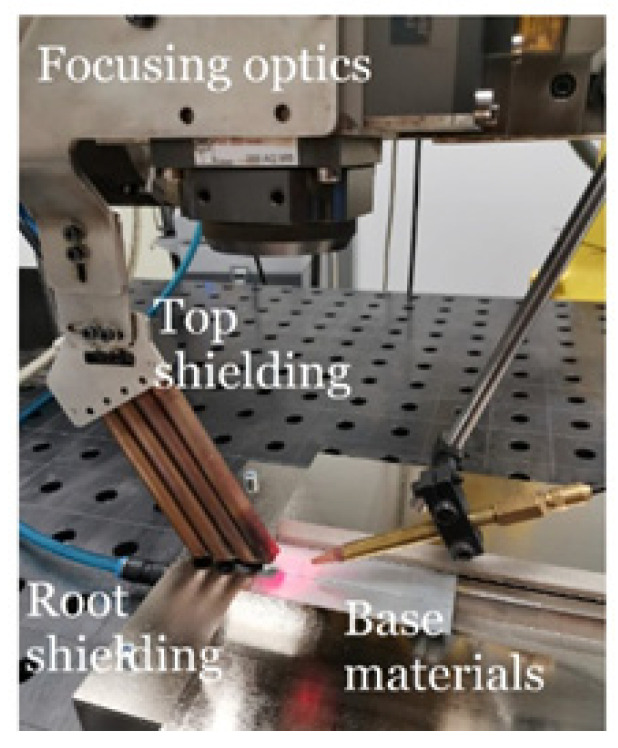
Set-up of materials to be welded.

Laser welding was performed with TruDisk 4002 disk laser (TRUMPF Pvt. Ltd., Pune-Maharashtra, India) with a maximum power of 2 kW and a wavelength of 1.03 μm (Figure 1). BEO D70 focusing optics was mounted on a 6-axis robot FANUC M-710iC/50 (FANUC Slovakia s.r.o, Nitra, Slovakia). Laser light cable with the core diameter of 400 μm was used for delivering laser radiation from the source to focusing optics. The spot size was 400 μm. Before welding, the sheet edges were adjusted after cutting on a CNC milling machine DMG MORI DMU 50. Samples were welded over the entire sheet width (800 mm) and length (1000 mm) in the PA (horizontal from the top) position in accordance with EN ISO 6947 without a gap between the sheets.

Welding parameters were chosen based on the preliminary experiments. Used welding parameters and quality evaluation methods are in Table 3.

**Figure 2:**
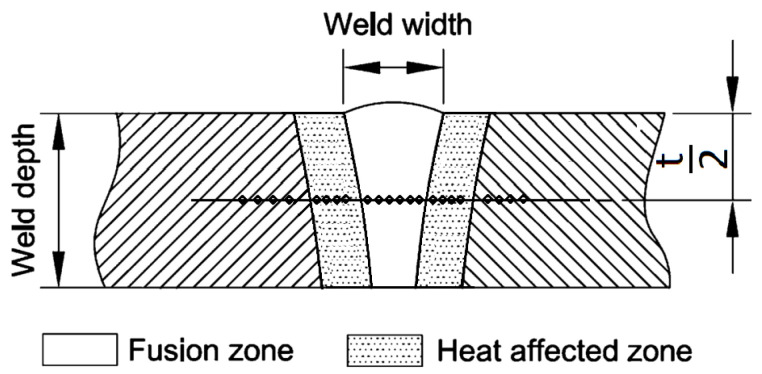
Scheme of microhardness testing.

Exemplary scheme of microhardness testing on area of the laser welded samples is demonstrated in Figure 2. The measurements were done in the depth that is half of the total thickness of each of the samples.

## 3. Results

Analysis of the quality of welded joints by visual inspection did not show the presence of external surface defects. However, a difference in thicknesses was noted between the base material and the joint. It is documented on macrostructures.

**Figure 3:**
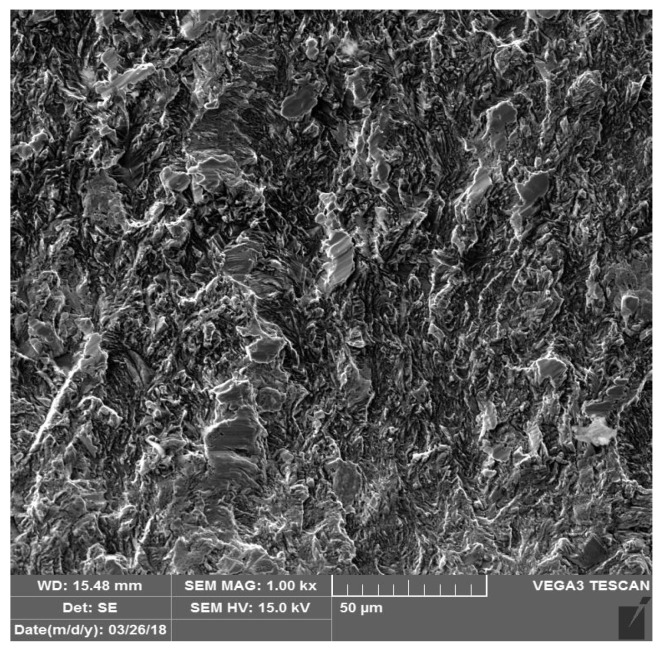
Fusion zone fractography after tensile test on sample C (TRIP).

According to the results of destructive tests for the relevant evaluation methods, which are given in Summary Table 4. The presented values are the arithmetic mean of the five measurements for each sheet type. It can be observed that samples C showed maximum load-bearing values, where the following average value of YS (yield strength) 448 ± 4 MPa and UTS (ultimate tensile strength) were measured 764 ± 4 MPa. This result is in accordance with the values given by the manufacturer. The lowest values of bearing capacity of welded joints were measured in samples A. In these samples the average values of yield strength 319 ± 4 MPa and tensile strength values 422 ± 4 MPa were measured. During the uniaxial tensile test, a fracture occurred in the base material in all evaluated samples. Observation of the fracture surface SEM after the tensile test on the fusion zone of the TRIP alloy of Al-alloyed steel shows a shear fracture shown in Figure 3. It is a fission quarry with minimal signs of plasticity. The surface of the fission veneers is not smooth, but contains numerous wrinkles, which are the result of, e.g., plastic deformation that precedes the formation or growth of a crack, the presence of grain boundaries or sub grains.

**Figure 4:**
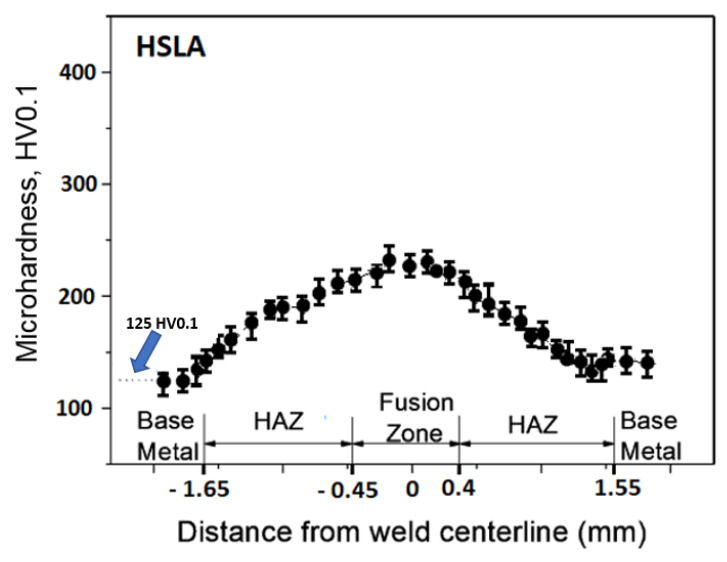
Weld microhardness profiles sample A (HSLA).

**Figure 5:**
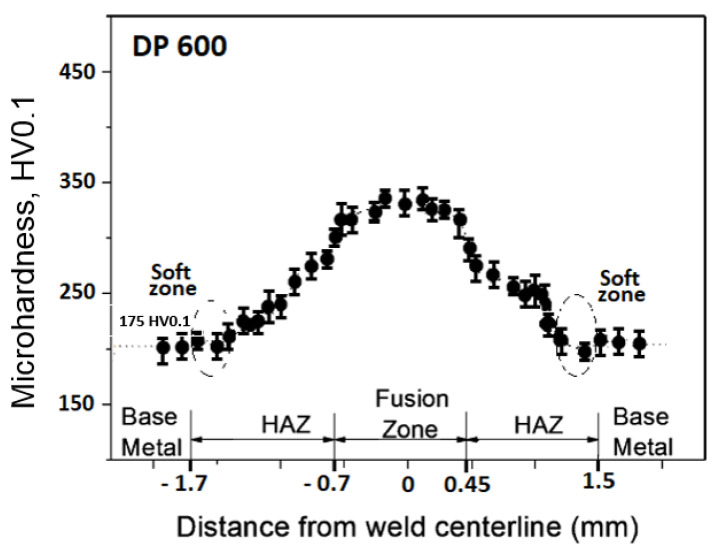
Weld microhardness profiles sample B (DP600).

**Figure 6:**
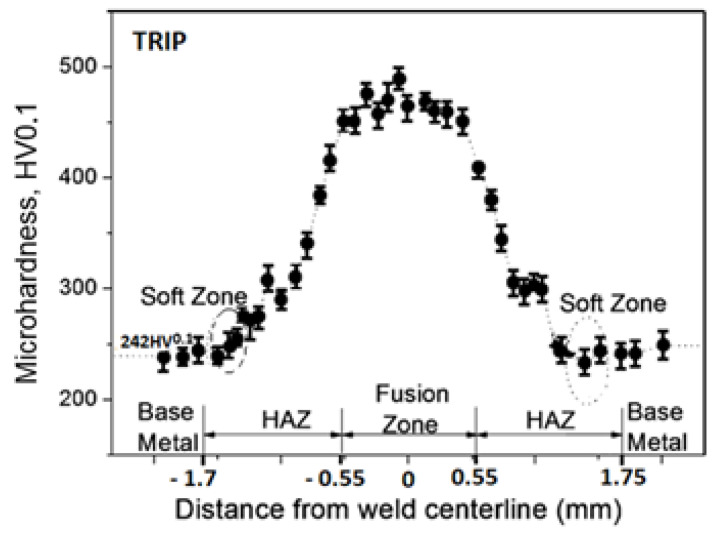
Weld microhardness profiles sample C (TRIP).

The measured microhardness values correspond to a chemical composition of an investigated materials and spotted structures. The lowest microhardness values were shown in the base material, samples A Figure 4, where the average microhardness value in the base material was 125 HV0.1, in thermally affected area the microhardness values raised to 179 HV0.1 and the weld metal showed an average microhardness of 231 HV0.1. The microhardness course of sample B is documented in Figure 5. On sample B (Figure 5) the lowest microhardness value in the base material 175 HV0.1 was measured. Microhardness peaks were recorded in the middle of the weld metal at 347 HV0.1. Samples marked C showed the maximum microhardness values. The average microhardness value of the base material was 242 HV0.1, the average value of 369 HV0.1 was measured in thermally affected area and the maximum values of 498 HV0.1 were measured in the weld metal (Figure 6).

**Figure 7:**
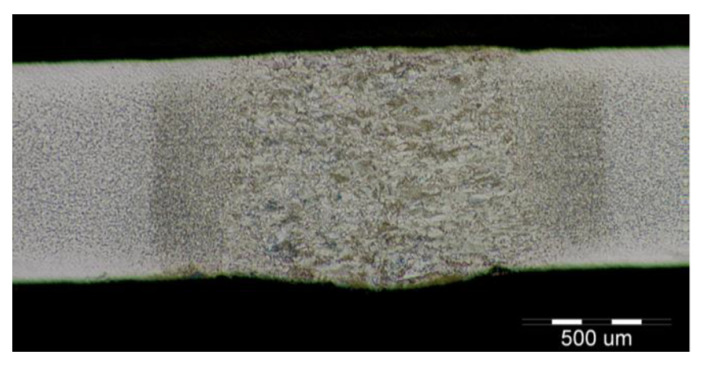
Macrostructure of sample A welded joint (HSLA).

**Figure 8:**
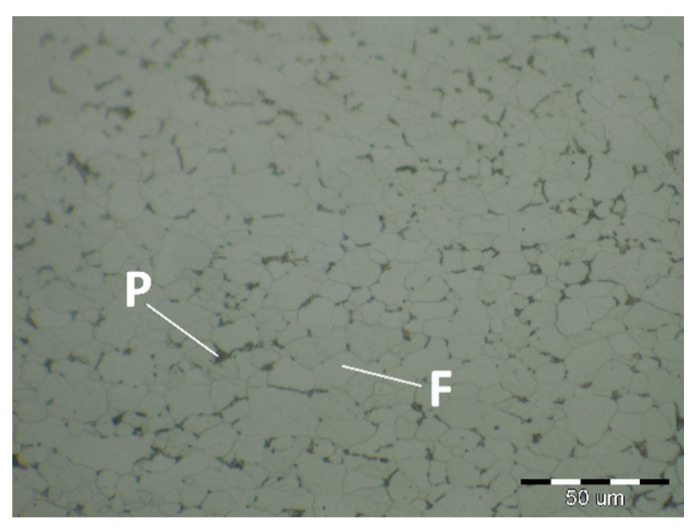
Base material microstructure—ferritic-pearlitic (HSLA).

**Figure 9:**
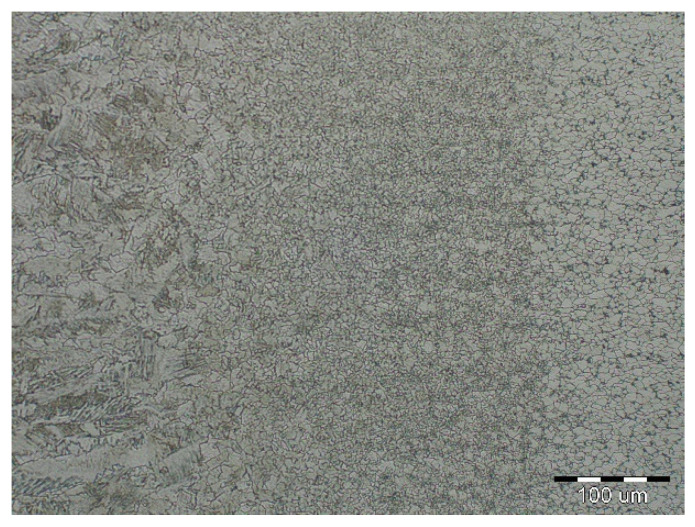
Microstructure of a narrow thermally affected area with a characteristic change in grain size in the recrystallization zone (HSLA).

**Figure 10:**
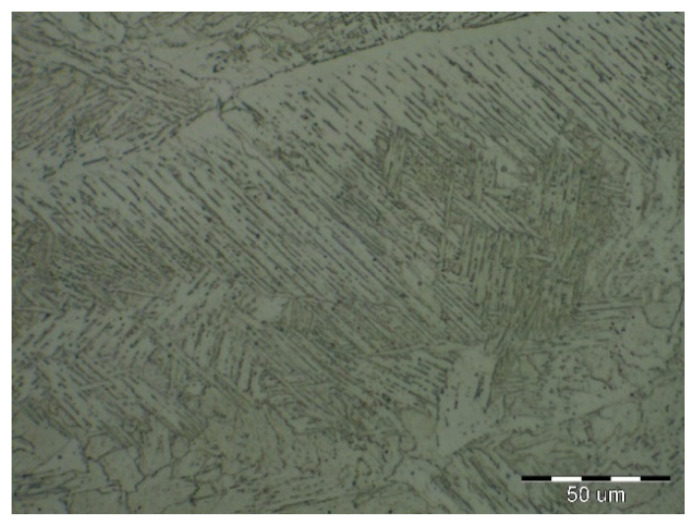
Coarse-grained microstructure of HSLA steel in the middle of the weld metal.

The fluctuations in the microhardness are the result of the high heat input since the forming of the joint involves many heat-activated phenomena including grain recovery and recrystallization. Analysis of macroscopic metallographic sections confirms the results of visual inspection of welded joints. The surface of the weld metal, which is made by laser, had a distinctive pattern with a well-readable welding direction. Structural analysis was performed by light cross-sectional microscopy. The macrostructure of the welded joint of sample A after etching is documented in (Figure 7). During etching, the width of the weld metal and the width of the heat-affected zones are clearly legible. The microstructure of the base material (Figure 8) is fine-grained with an average grain size of G7 according EN ISO 643:2019 standard, which consists of polyhedral ferrite and perlite in a volume of maximum 10%. The volume ratio of polyhedral ferrite to perlite was determined using QuickPHOTO MICRO—Microscope Software. In the narrow heat affected zone, a change in grain size and transformation of the base structure into transformed fluffy perlite and acicular ferrite were observed. This is consistent with the measured microhardness values in this area (Figure 9). The weld metal has a typical coarse-grained structure (G4). Which consists primarily of a decaying mixed structure of acicular ferrite and secondarily polygonal ferrite (Figure 10).

**Figure 11:**
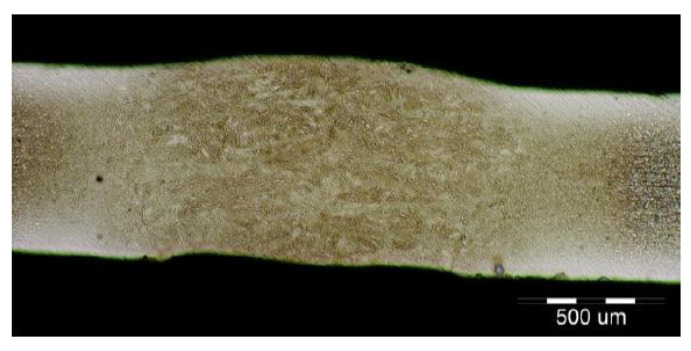
Macrostructure of the welded joint sample B (DP600).

**Figure 12:**
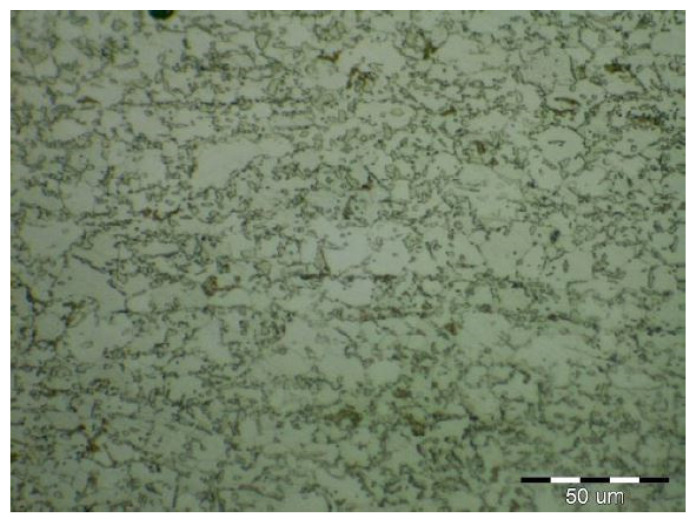
Two-phase microstructure of DP 600 base material.

**Figure 13:**
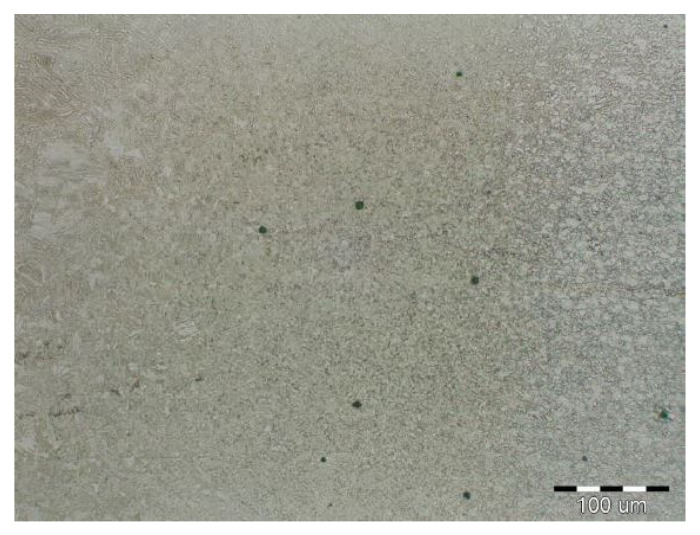
Microstructure of thermally affected area sample B (DP600).

The formation of different structures is influenced by various factors, namely the number of alloying elements, the rate of cooling, the presence of oxygen in the weld and the grain size of the previous austenitic grains. The cooling rate contributes to other factors in the final formation of the microstructure of laser-welded steels. The macrostructure of the welded joint of sample B made by the laser is shown in Figure 11. Only a small overhang of the weld is recorded on the macrostructure. The structure of the base material made of DP 600 steel (Figure 12) consists of a ferritic matrix in which martensite is dispersed in a volume of approx. 15%. The average grain size of the ferritic matrix was G7 according to EN ISO 643:2019 standard. The martensitic phase ensures an increase in the strength of the steel. This is necessary for the passive safety of cars, especially in deformation zones. Such properties are currently the most sought after manufacturers of body parts and these types of steel are used in the manufacture of passenger cars. The heat affected zone is formed by a multiphase structure (Figure 13). In the “soft area” immediately adjacent to the base material, there is a fine-grained microstructure of tempered martensite and bainite in a ferritic matrix. Fine-grained residual austenite as well as spherical inclusions were observed in the structure. Towards the weld, the grains increased due to the introduced heat. These were increased by the proportion of martensitic-bainitic phase, which also corresponds to the measured microhardness values.

**Figure 14:**
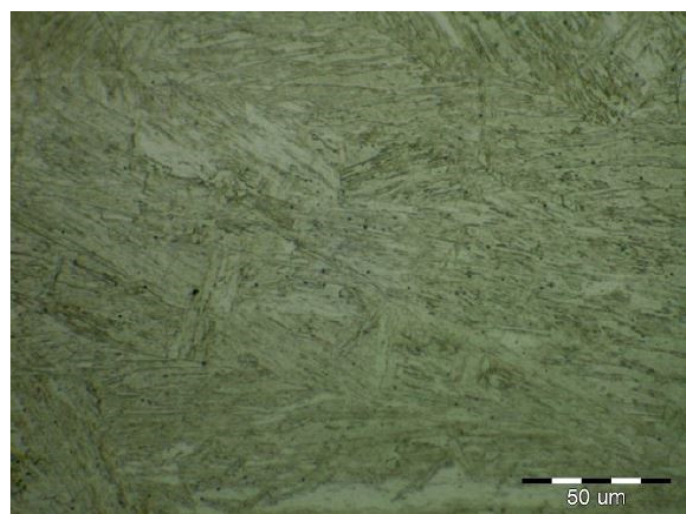
Weld metal microstructure of sample B (DP600).

**Figure 15:**
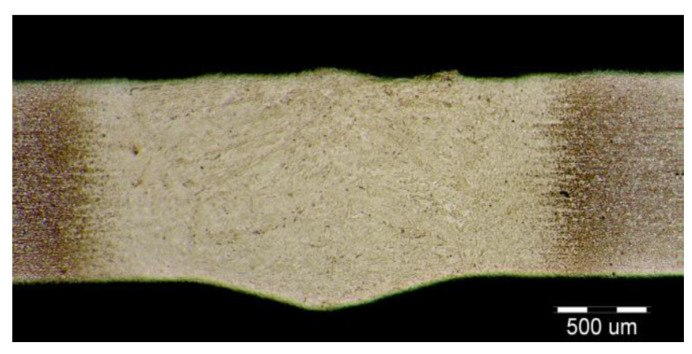
Macrostructure of a welded joint. Samples C (TRIP).

**Figure 16:**
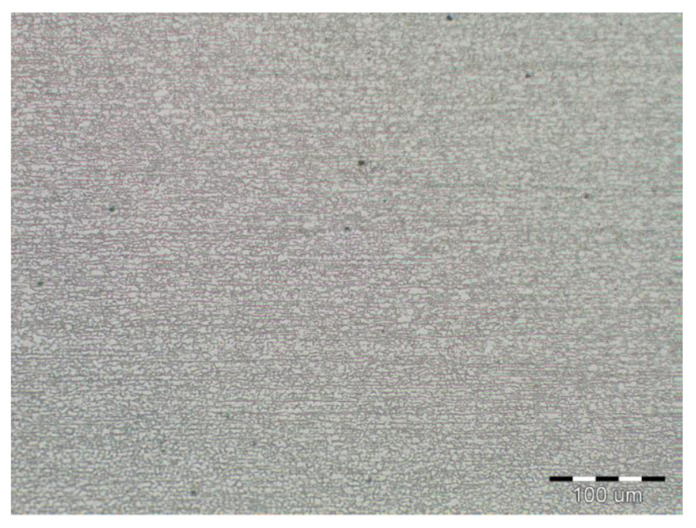
Fine-grained multiphase microstructure of TRIP base material.

**Figure 17:**
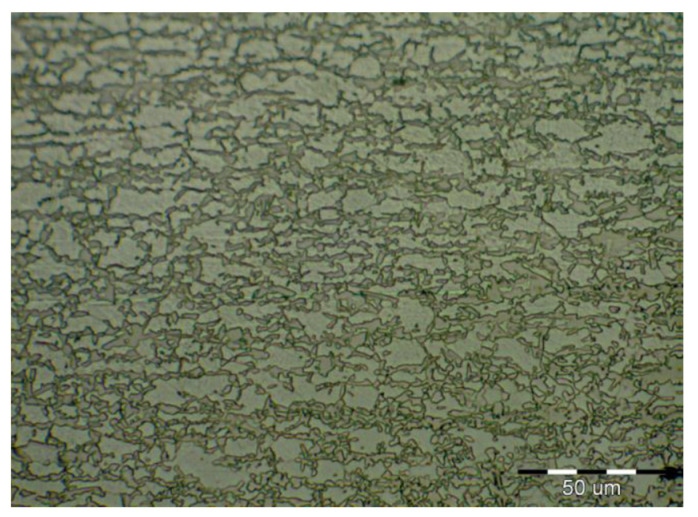
Detail of the TRIP base material microstructure.

Due to the rapid weld pool cooling, the weld metal structure (Figure 14) consists mainly of coarse-grained lamellar martensite, bainite and the rare acicular ferrite. The welded joint macrostructure, sample C, TRIP steel sheets is shown on Figure 15. The laser weld has a characteristic drawing on the cut with a readable dihedral culinary crystals angle. The weld metal is slightly elevated. Macroscopic analysis did not show the presence of pores, cavities, and internal defects. These occur quite often in the middle of weld lenses in resistance spot welding with this type of material. The weld area aswell as the thermally affected area are narrow. The base material has a fine-grained structure with an average grain size of G9 according to EN ISO 643:2019 standard. The TRIP steel microstructure (Figure 16 and Figure 17) is multiphase formed of polyhedral ferrite, martensite, bainite and residual austenite. Carbon equivalent (CE) is evaluated with Yurioka formula as follows [74]:

CE = C + ƒ(*C*) {Si/24 + Mn/6 + Cu/15 + Ni/20 + (Cr + Mo + Nb + V)/5} (1)

where ƒ(*C*) = 0.75 + 0.25 tanh {20(C − 0.12)}.

The measured microhardness of fusion zone (498 HV0.1) is even higher than the martensite microhardness (370 HV0.1) calculated with the Yurioka formula as follows [71].

*H_M_* = 884*C* + 294 (2)

**Figure 18:**
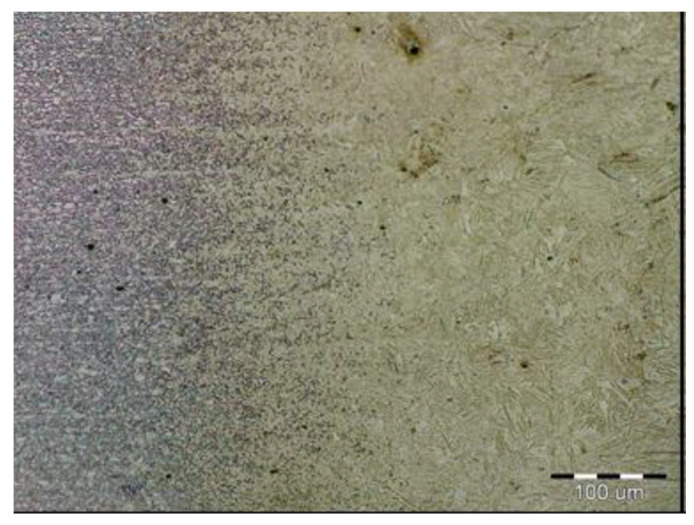
Narrow transition area base material to thermally affected area (TRIP).

**Figure 19:**
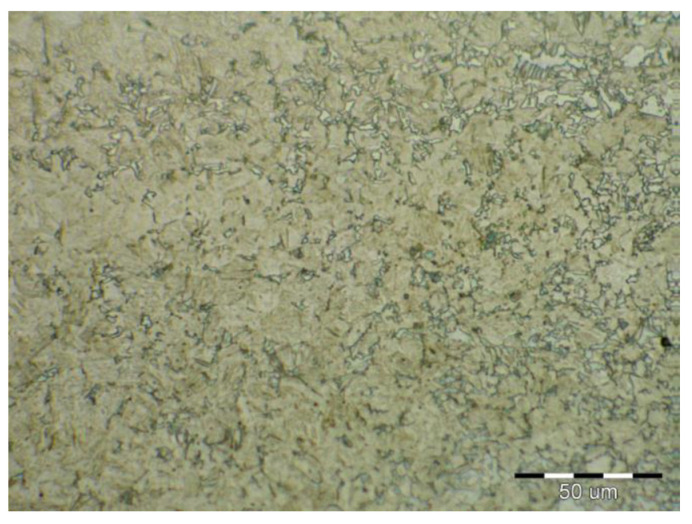
Thermally affected area microstructure formed by martensite and ferrite (TRIP).

**Figure 20:**
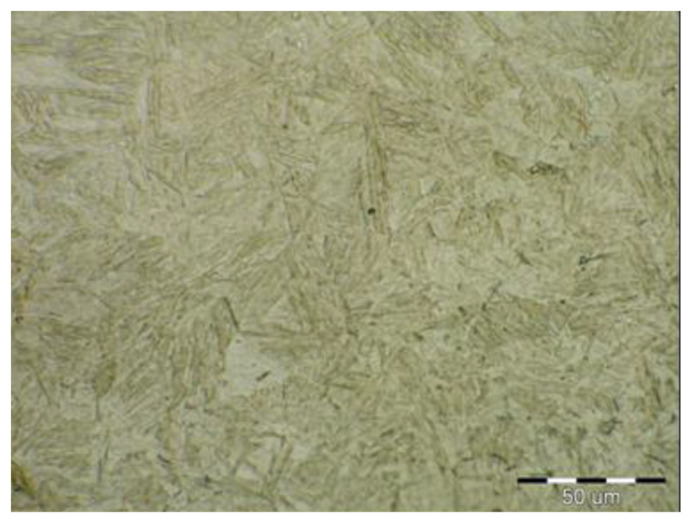
Microstructure in the middle of weld metal area formed by martensite and bainite (TRIP).

The structure is formed by fine-grained ferrite and martensite in the narrow heat-affected region of the laser weld seen in Figure 18 and Figure 19. The coarse-grained structure of martensite and bainite was recorded in the middle of the weld metal (see Figure 20). The weld metal of the multiphase structure of the steel primarily alloyed with Al has a microhardness of 498 HV0.1, which significantly exceeds the maximum calculated value of hardness (370 HV0.1) according to Equation (2). To increase the contrast between the ferrite and the individual phases in Figure 16, a 5% nital etchant was used for 15 s. The literature [75] states that the decisive role in the formation of the resulting structure in TRIP steel is played by Al resp. Si which slow down the precipitation of carbides in steel. The addition of Al intensively stabilizes the ferrite and promotes the precipitation of high-temperature ferrite as the primary phase in the solidification process. The presence of vermicular ferritic as well as skeletal ferritic morphology, which occurs at a solidification rate of up to 103 K/s, has been reported in the weld metal [76]. The presence of skeletal ferrite in the weld metal is a remnant of high-temperature delta ferrite, which did not convert to austenite in the entire volume during cooling. In a study [77], the precipitation of fine lamellar ferrite plates along bainite-martensite between skeletal ferrite was noted. Residual austenite plays two important roles. The first primary is similar to the addition of Al in the processes of precipitation of carbide phases and influencing the rate of these processes [78]. The secondary where it provides the TRIP effect in the fusion region by transformation into martensite and under stress contributes to the required uniform elongation of TRIP steels.

## 4. Conclusions

The main findings of this research are related to the development of laser welding technology, as well as metallurgy methods, which take into account phase and structural transformations that occur in the weld and in its vicinity during laser welding, in particular:

The distribution of microhardness in laser welds made in all investigated steels (HSLA, DP600, and TRIP) was found to be symmetrical relative to the weld axis. In addition, the weld microhardness (HV0.1) was found to attain its maximum in the fusion zone for all investigated steels: for HSLA steel (HV0.1 = 240), for DP600 steel (HV0.1 = 347), for TRIP steel (HV0.1 = 498). The highest value of microhardness was shown to correspond to the weld axis, which is associated with the ferritic-martensitic structure present in the crystallization zone. A decrease in microhardness was observed at some distance from the weld axis, the minimum value of which corresponds to the microhardness of the cold-rolled base metal.

The width of the welding zone for steels HSLA, DP600 and TRIP was 0.85, 1.1 and 1.1 mm respectively. An increase in the weld zone width causes an increase in the structural heterogeneity of the material in the adjacent zones, which is reflected in the distribution of microhardness values. Hardness measurements showed the presence of a very narrow soft zone in the heat affected zone (HAZ) adjacent to the weld interface, which does not affect the tensile strength of the weld. During static tensile test of welds, all test specimens got fracture in the base metal, indicating the uniformity of weld microstructure in steels studied.

The findings of research on laser welding of steels will aid in the automation of various technological processes of laser heat treatment. In addition, they can be useful in the welding of car bodies, leading to an increased production efficiency due to additional quality control of parts in real time.

## Figures and Tables

**Table 1:** Chemical composition of welded steel sheets (wt %).

A-HSLA	C	Mn	Si	P	S	Al	Cu	Cr
	0.005	0.409	0.128	0.037	<0.002	0.034	0.015	0.031
	Mo	Ni	V	Ti	Nb	Co	W	Fe
	0.008	0.006	0.006	0.033	0.035	0.021	0.038	Res.
B-DP600	C	Mn	Si	P	S	Al	Cu	Cr
	0.111	1.963	0.279	0.026	<0.002	0.031	0.019	0.206
	Mo	Ni	V	Ti	Nb	Co	W	Fe
	<0.002	<0.002	0.012	<0.002	0.020	0.017	0.005	Res.
C-TRIP	C	Mn	Si	P	S	Al	Cu	Cr
	0.087	1.487	0.184	0.024	<0.002	2.245	0.021	0.041
	Mo	Ni	V	Ti	Nb	Co	W	Fe
	0.022	<0.002	0.011	0.007	0.023	0.022	<0.002	Res.

**Table 2:** Mechanical properties of the evaluated sheets specified by the manufacturer.

Steel	Yield Strength (MPa)	Ultimate Tensile Strength (MPa)	Elongation A_5_ (%)
A-HSLA	240–330	340–450	32
B-DP600	300–470	580–670	26
C-TRIP	480	min. 700	25

**Table 3:** Used welding parameters and welded joints evaluation methods.

Welding Parameters:	
Laser power	1200 W
Laser mode	Multi-Mode
Shielding gas	High Purity: Ar 4.8—Argon 99.998%
Flow rate Ar	20 L/min
Welding speed	2.4 m/min
Laser beam focusing	On the surface of the sheet f = 0 mm
Wavelength	λ = 1.03 μm
Methods of evaluation of welded joints:	Standard:
The visual testing of fusion welds in metallic materials.	ISO 17637
Tensile tests of a welded joint in the transverse direction.	EN ISO 4136,
Microhardness testing on transverse sections of welded joints of metallic materials.	EN ISO 9015-2
Macroscopic and microscopic examination of welds by Olympus BXFM light microscope.	EN ISO 17639
For etching and macro and microstructures visualization the nital (2% HNO_3_ solution) was used	-

**Table 4:** Average values of experimental steel tensile properties.

Sample	Yield Strength (MPa)	Ultimate Tensile Strength (MPa)	Failure Location
A-HSLA	319 ± 4	422 ± 4	Base material
B-DP600	372 ± 3	620 ± 5	Base material
C-TRIP	448 ± 4	764 ± 4	Base material

## Data Availability

The data presented in this study are available on request from the corresponding author. The data are not publicly available due to their extended size, incompatible with online upload.
